# Case Report: Early-Onset Behavioral Variant Frontotemporal Dementia in Patient With Retrotransposed Full-Length Transcript of *Matrin-3* Variant 5

**DOI:** 10.3389/fneur.2020.600468

**Published:** 2020-12-21

**Authors:** Madelyn Castro, Nisha Venkateswaran, Samuel T. Peters, David R. Deyle, Matthew Bower, Michael D. Koob, Bradley F. Boeve, Keith Vossel

**Affiliations:** ^1^Department of Neurology, N. Bud Grossman Center for Memory Research and Care, University of Minnesota, Minneapolis, MN, United States; ^2^Department of Neurology, Mary S. Easton Center for Alzheimer's Disease Research at UCLA, David Geffen School of Medicine at UCLA, Los Angeles, CA, United States; ^3^Department of Clinical Genomics, Mayo Clinic Rochester, Rochester, MN, United States; ^4^Division of Genetics and Metabolism, University of Minnesota, Minneapolis, MN, United States; ^5^Molecular Diagnostics Laboratory, M Health-Fairview, University of Minnesota, Minneapolis, MN, United States; ^6^Department of Laboratory Medicine and Pathology, University of Minnesota, Minneapolis, MN, United States; ^7^Department of Neurology, Mayo Clinic Rochester, Rochester, MN, United States; ^8^Institute for Translational Neuroscience, University of Minnesota Medical School, Minneapolis, MN, United States

**Keywords:** frontotemporal dementia, Matrin 3, case report, retrotransposons, whole genome sequencing

## Abstract

Frontotemporal dementia (FTD) rarely occurs in individuals under the age of 30, and genetic causes of early-onset FTD are largely unknown. The current report follows a 27 year-old patient with no significant past medical history presenting with two years of progressive changes in behavior, rushed speech, verbal aggression, and social withdrawal. MRI and FDG-PET imaging of the brain revealed changes maximally in the frontal and temporal lobes, which along with the clinical features, are consistent with behavioral variant FTD. Next generation sequencing of a panel of 28 genes associated with dementia and amyotrophic lateral sclerosis (ALS) initially revealed a duplication of exon 15 in *Matrin-3* (*MATR3*). Whole genome sequencing determined that this genetic anomaly was, in fact, a sequence corresponding with full-length *MATR3* variant 5 inserted into chromosome 12, indicating retrotransposition from a messenger RNA intermediate. To our knowledge, this is a novel mutation of *MATR3*, as the majority of mutations in *MATR3* linked to FTD-ALS are point mutations. Genomic DNA analysis revealed that this mutation is also present in one unaffected first-degree relative and one unaffected second-degree relative. This suggests that the mutation is either a disease-causing mutation with incomplete penetrance, which has been observed in heritable FTD, or a benign variant. Retrotransposons are not often implicated in neurodegenerative diseases; thus, it is crucial to clarify the potential role of this *MATR3* variant 5 retrotransposition in early-onset FTD.

## Introduction

Frontotemporal dementia (FTD) refers to a group of dementias characterized by degeneration in the frontal and temporal lobes of the brain (1). Rates of early-onset FTD are high with 13% of individuals with FTD being under the age of 50 (2). Younger cases of FTD, with onset before the age of 30, tend to exhibit frequent abrupt mood changes, increased aggression, behavioral disinhibition, lack of empathy, and deficits in working memory (3). There are three clinical presentations of FTD: behavioral variant (bvFTD) and two forms of primary progressive aphasia, wherein non-fluent or fluent aphasia are the key neurologic deficits (4, 5). In addition, FTD can overlap with other neurodegenerative disease motor deficits including: corticobasal degeneration, progressive supranuclear palsy, and amyotrophic lateral sclerosis (ALS) (6). Approximately 30–50% of FTD cases have some family history of dementia, parkinsonism or ALS (7), and in 10–20% a genetic cause is found (8). Positive family history of dementia/parkinsonism/ALS typically shows an autosomal dominant pattern with high penetrance, with at least one first-degree relative of the proband being affected (7). Mutations in genes encoding microtubule associated protein tau (*MAPT*), progranulin (*GRN*), and chromosome 9 open reading frame 72 (*C9orf72*) account for about half of all familial cases of FTD (9, 10). Mutations in *MATR3*, which encodes matrin-3, have been found in some cases of familial FTD as well (11). Matrin-3 is a DNA- and RNA-binding protein that is part of the nuclear matrix and has a wide variety of functions including transcriptional regulation, DNA binding, and RNA splicing and degradation (11, 12). Matrin-3 interacts with pathologic markers, such as TAR DNA-binding protein 43 (TDP-43), which aggregates in neuronal cytoplasm in both FTD and ALS (13).

There are several pathological subtypes of FTD, categorized under frontotemporal lobar degeneration (FTLD) and classified by the presence of abnormal components of neuronal and glial inclusions. Some of the major subtypes include microtubule-associated protein tau in FTLD-tau, TDP-43 in FTLD-TDP, and fused in sarcoma (FUS) protein in FTLD-FUS (14). FTLD-TDP is the most common pathological subtype, occurring in ~50% of all cases. FTLD-tau accounts for ~45% of all cases, followed by FTLD-FUS in <5% of cases (13). The majority of individuals with FTLD-FUS are diagnosed with sporadic, early-onset bvFTD (6).

This report discusses the clinical presentation and genetic findings of an individual with early-onset FTD. Collectively, FTD is clinically heterogeneous, making it difficult to predict the underlying pathological or genetic processes. Thus, with these findings we aim to further understand the molecular pathology and systemic features of early-onset forms of FTD.

## Case Report

The patient was in good baseline health until age 27 years, when her family noticed an increase in aggression and child-like behaviors. Initially, a family member brought her to the emergency department for behaving erratically. She was later discharged and diagnosed with adjustment disorder with mixed emotional features.

Four months later, the patient was brought into the emergency department (ED) with suspicion of a psychiatric disorder. On exam, she was noticeably withdrawn and inattentive. Due to apparent psychosis, she was placed on hold and hospitalized. Following admission, she was given a provisional diagnosis of bipolar disorder type 1. She was placed on oral olanzapine and observed over three days with slight improvements in symptoms allowing for voluntary discharge.

**Figure 1:**
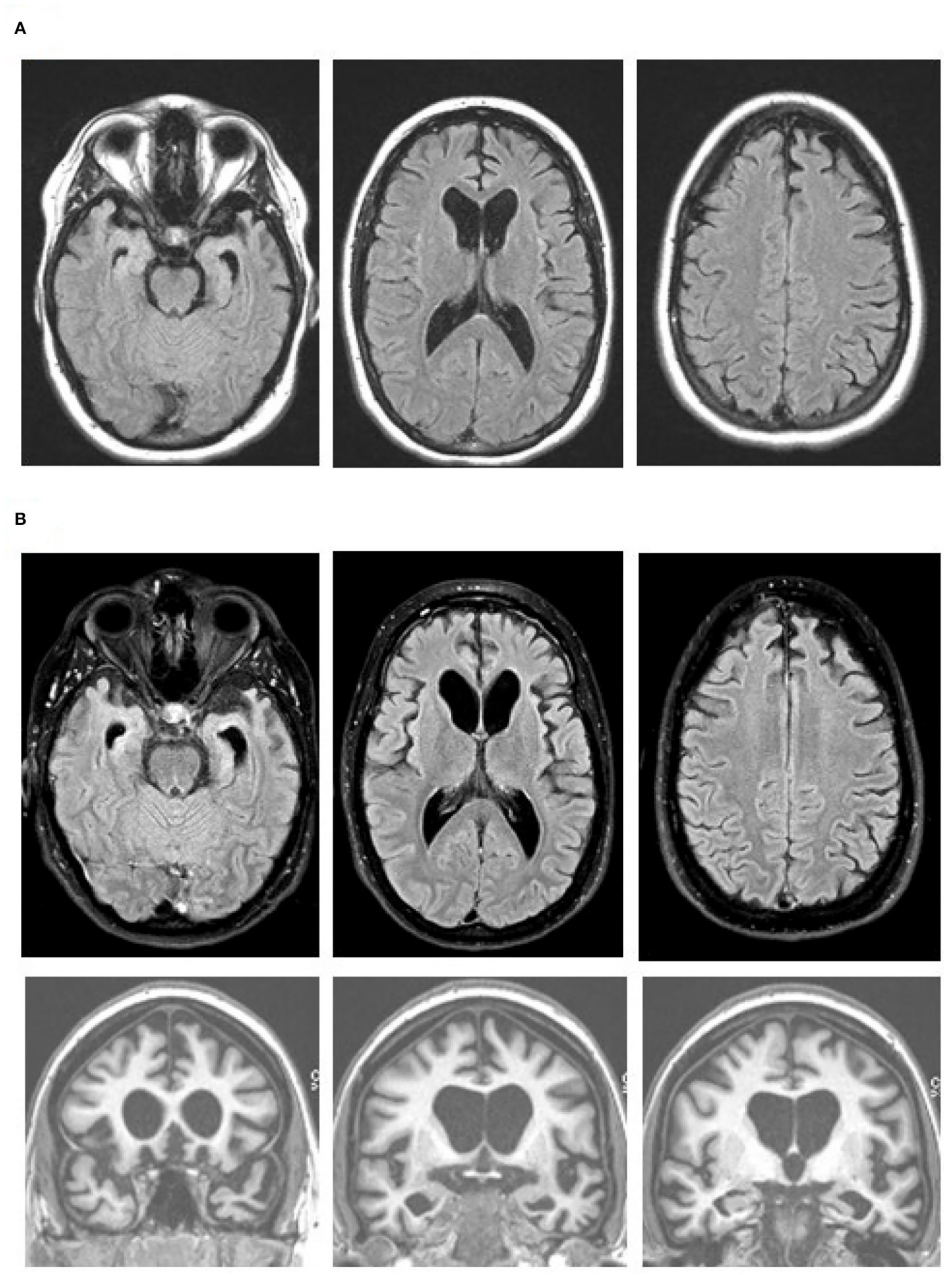
Brain MR images at age 27 **(A)** and 19 months later **(B)**, with axial fluid attenuation inversion recovery (FLAIR) images (top and middle rows) and coronal T1-weighted images (bottom row). Note progression of the bilateral medial frontal, anterior temporal, and caudate head atrophy with the associated ventricular enlargement on the axial FLAIR images. The orbitofrontal as well as dorsomedial, anterior temporal, and caudate head atrophy are obvious on the coronal images. Images are in standard radiology orientation.

The patient was brought to the ED again two months later after a physical altercation. In the ED, her psychiatric exam was notable for disorganized thoughts, mood lability, poor insight, and rapid, tangential speech; however, she was not agitated or aggressive. She was admitted on a 72-h hold for mania and psychosis. Her drug screen was negative and she was admitted to the psychiatry service. She did not respond to multiple antipsychotic medications or neuroleptic drugs. For workup of medically refractory psychosis, she completed a brain MRI which showed bilateral medial frontal, anterior temporal, and caudate head atrophy (Figure 1A). Clinicians in the neurology department then evaluated her and transferred her to their service for further testing.

She completed an EEG, MRI, and lumbar puncture. The EEG was characterized by diffuse theta/delta slowing consistent with non-specific encephalopathy of unknown etiology. Her second MRI, two months after initial MRI, showed similar changes to the prior MRI. The cerebrospinal fluid workup for infectious and autoimmune conditions was unrevealing. The cerebrospinal fluid autoantibody panel inclued NMDA-R, CASPR2-lgG, GAD65, ANNA-3, AGNA-1, PCA-Tr, Amphiphysin, VGKC-Complex, LGI1-lgG, GABA-B-R, AMPA-R, ANNA-1, ANNA-2, PCA-1, PCA-2, and CRMP-5-IgG. The blood autoantibody panel included NMDA-R, CASPR2-lgG, GAD65, GABA-B-R, AMPA-R, ANNA-1, ANNA-2, ANNA-3, AGNA-1, PCA-Tr, Amphiphysin, ACh Receptor (Muscle) Binding, AChR Ganglionic Neuronal, Neuronal (V-G) K+ Channel, LGI1-lgG, PCA-1, PCA-2, N-Type Calcium Channel, P/Q-Type Calcium Channel, and CRMP-5-IgG. After completing these exams, she was readmitted to the psychiatry department.

**Figure 2:**
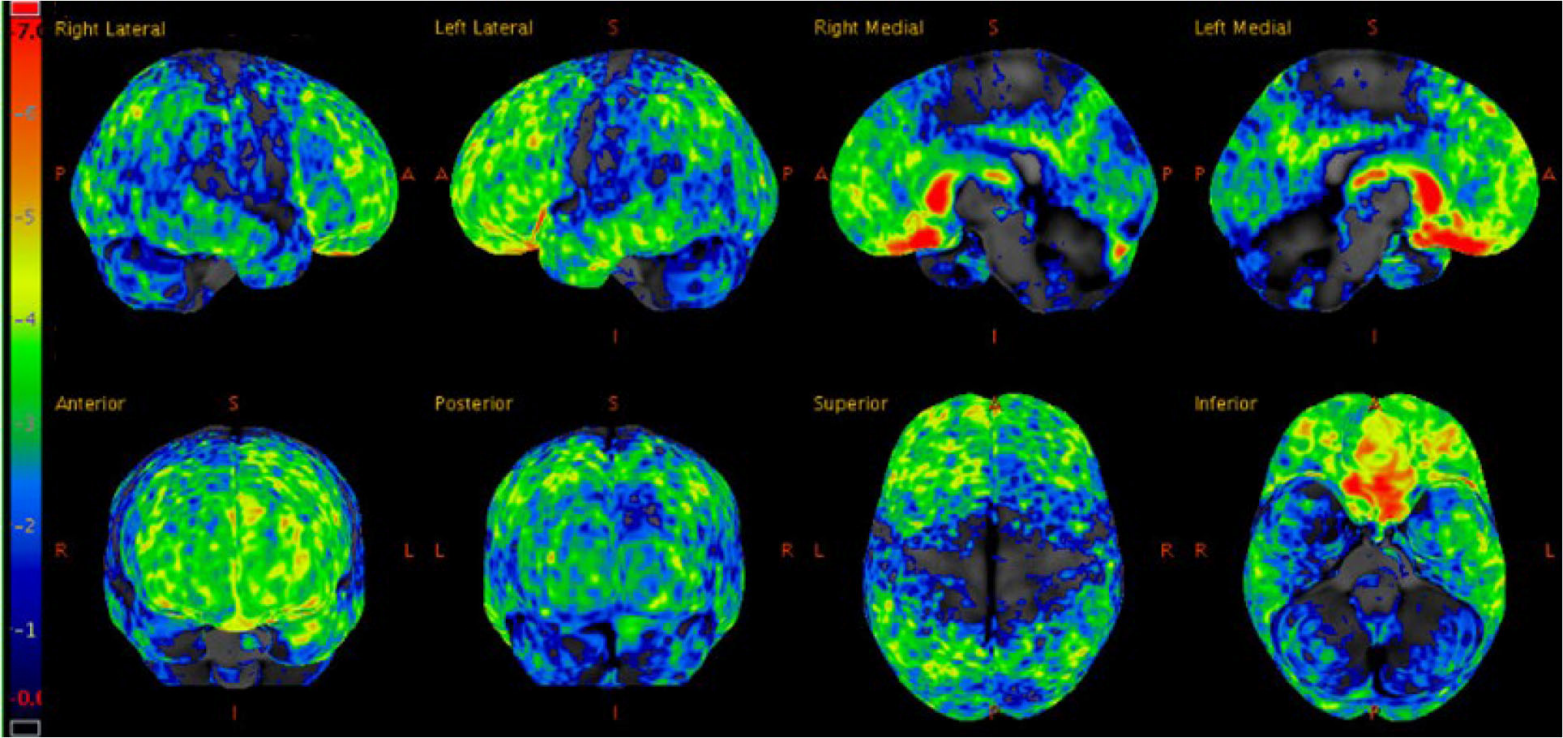
Statistical z-score maps of brain FDG-PET at age 27, with the degree of hypometabolism reflected by the color bar on the left; dark blue is within normal limits and red is maximally abnormal. Note the degree of hypometabolism is severe in the orbitofrontal and caudate regions, moderate in the frontotemporal regions (left slightly more so than right), and mild to moderate in the parietal and posterior cingulate regions.

Three weeks later, a fluorodeoxyglucose (FDG)-positron emission tomography (PET) scan was performed, which showed hypometabolism that was severe in the orbitofrontal and caudate regions, moderate in the frontotemporal regions, and mild to moderate in the parietal and posterior cingulate regions (Figure 2). FDG-PET from skull to thigh revealed no evidence of malignancy. In the following months, her behavioral disinhibition and psychosis did not improve on multiple antipsychotic medications. She was administered six courses of electroconvulsive therapy over two weeks but showed no improvement.

Although a primary psychiatric illness was considered on initial presentation, her clinical features, lack of response to antipsychotics, and prominent atrophy seen via brain imaging indicated a neurodegenerative condition. The brain MRI and FDG-PET findings, along with her clinical features were consistent with behavioral variant FTD (bvFTD) (14). Her presentation was notable for lack of insight, diminished empathy, aberrant motor behaviors, verbal aggression, stereotyped phrases, social withdrawal, disinhibition, and environmental dependence. There was no clinical evidence of motor or neuromuscular dysfunction. A five generation family history was reviewed and, with the exception of one second-degree relative with suspected mild cognitive impairment, two second-degree relatives with developmental delay, and one third-degree relative with arachnoid cyst, no other contributory or signficant family history was noted. Given the extremely young age and aggressive behavior with maintained verbal fluency, lack of family history of a neurodegenerative disease, and imaging findings particularly with prominent caudate atrophy/hypometabolsim, bvFTD with FUS proteinopathy was considered a strong possibility. MRI performed 19 months after the initial scan showed a progression of the medial frontal, anterior temporal, and caudate head atrophy with the associated ventricular enlargement (Figure 1B). No restricted diffusion was seen on this MRI or previous MRIs.

## Genetic Analysis

Due to the unique presentation, we performed next generation sequencing (NGS) to analyze a panel of 28 genes associated with dementia and ALS. The panel included *ALS2, APP, CHCHD10, CHMP2B, PCTN1, FUS, GRN, HNRNPA2B1, MAPT, MATR3, OPTN, PFN1, DRNP, PSEN1, PSEN2, PSG11, SETX, SIGMAR1, SNCA, SOD1, SQSTM1, TARDBP, TBK1, TFG, TREM2, UBQLN2, VAPB*, and *VCP*. Genetic analysis initially indicated a duplication of exon 15 from the *MATR3* gene in the patient. NGS of both parents' DNA revealed this same feature in one clinically unaffected parent. All other gene tests in this panel were negative. We also assessed for hexanucleotide repeat expansion in *C9orf72* in the proband using a PCR based assay, and this test was normal (<20). In addition, we did clinical whole exome sequencing on the patient and the parents to evaluate for any other possible causes of FTD, which was performed using the Agilent SureSelect NovaSeq instrument with average coverage across the entire capture of 180x. The clinical whole exome sequence was negative for the patient and the parents.

We obtained blood samples from the patient and the parents to further characterize the *MATR3* mutation that was identified through NGS. We performed whole genome sequencing (WGS) through the Genomics Center as follows. Three TruSeq unique dual-indexed DNA libraries were created. All libraries were pooled and sequenced on a NovaSeq 6000 system, with S2-type flow cell and run mode of 2 × 150 bp. ~300 million pass-filter reads or greater were generated for each sample. Mean quality scores for all libraries were ≥ Q30 indicating <0.1% error rate in base calling. WGS indicated that variant 5 *MATR3* cDNA (*MATR3V5*) had been inserted into chromosome 12 (CH12), flanked by 15 base-pair repeats, with an insertion site 20 kbp upstream of the LIM Homeobox 5 (*LHX5)* gene. WGS results refuted the presence of an exon 15 duplication on chromosome 5, which was not detected. Huntington's disease was ruled out by WGS indicating that there was no evidence for *HTT* CAG repeat expansion in any of the samples.

**Figure 3:**
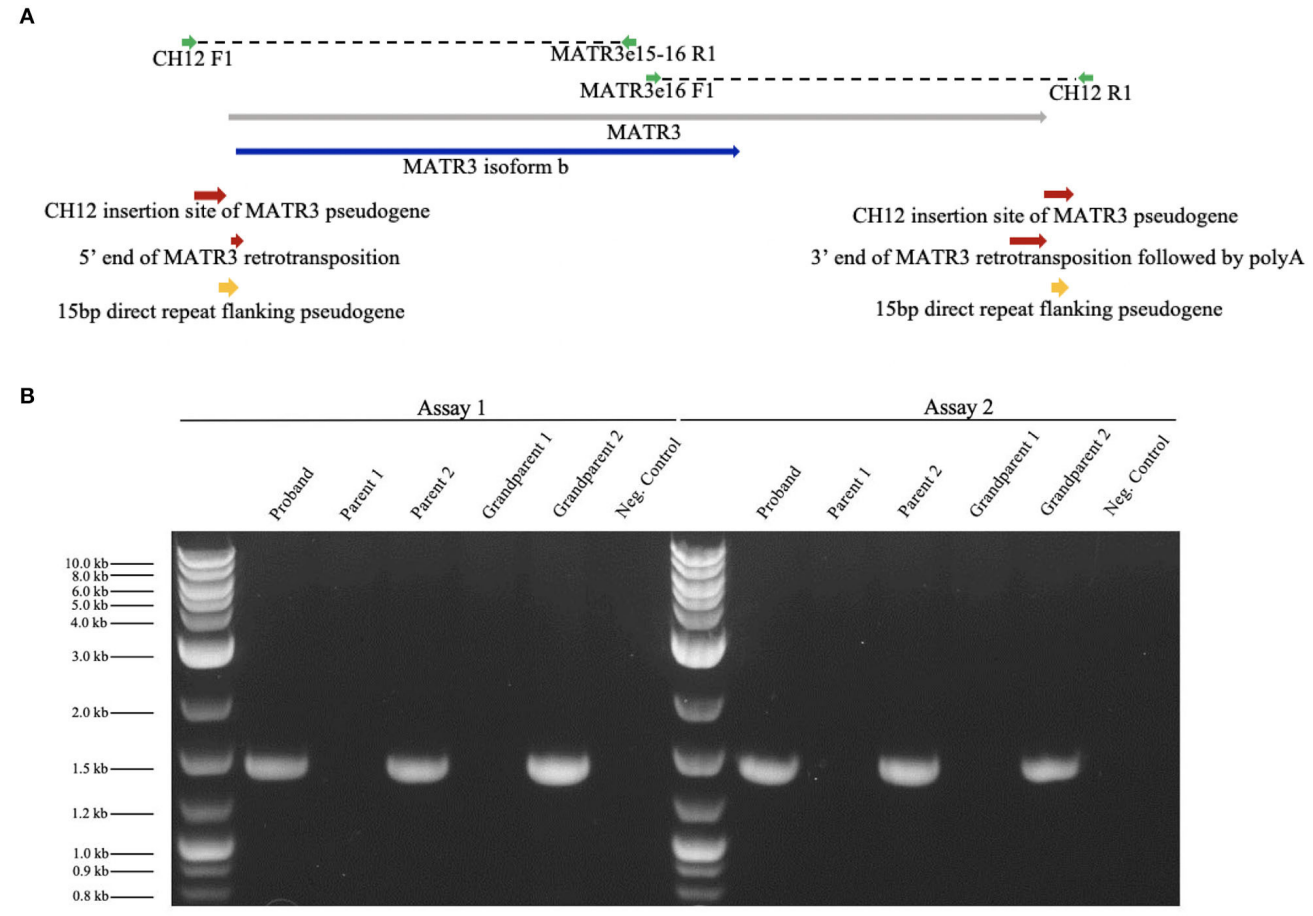
Targeted PCR confirmed the *MATR3V5* insertion in chromosome 12. **(A)** Diagram of chromosome 12 insertion site of *MATR3* variant 5 and primers used for PCR. *MATR3* gene sequence that codes for the matrin-3 protein isoform b is indicated in blue. **(B)** Patient (proband), parental, and grandparental genomic DNA were amplified by targeted PCR. Genomic DNA from the patient, parent 2, and grandparent 2 carried the mutation found in chromosome 12. A 1 kbp ladder was used in lanes 1 and 8. The expected product sizes were 1,457 bp for Assay 1 and 1,435 bp for Assay 2. Parent 1's genomic DNA was used for the negative control which lacked DNA polymerase.

We verified the results of WGS using Phusion High-Fidelity PCR (Thermo-Fisher) with primers crossing putative CH12/*MATR3V5* junctions. Two independent assays were completed: assay 1 [forward primer (CH12F1), TCTCTGCTGGCTCTACCTAAA; reverse primer (MATR3e15-16R1), AGTTCCTCGATCTTGTCCACC], and assay 2 [forward primer (MATR3e16F1), TGAGAACGCTGATGATCCCAA; reverse primer (CH12R1), AAAAAGGTGTTTCCTGGGAGCG], which targeted the 5′ and 3′ ends of the insertion, respectively (Figure 3A). Presence of DNA was confirmed and band size was measured using UV/ethidium bromide visualization in 1% agarose gel. The mutation was confirmed in the patient and one healthy parent (Figure 3B). To determine whether this mutation could have arisen *de novo* in the unaffected parent, blood and serum samples were collected from both grandparents biologically related to the healthy parent carrier. Targeted PCR revealed that one healthy grandparent also carries the *MATR3V5* insertion in CH12 (Figure 3B). These results indicate that, despite the *MATR3V5* mutation being present in multiple generations, only the patient showed clinical symptoms.

To search for *MATR3* transcripts in blood, total RNA was isolated using the PAXgene Blood RNA Kit for *in vitro* diagnostics (Qiagen, University of Minnesota Genomics Core). RNA integrity numbers were 7.4, 7.7, and 8.4 for the patient, parent, and grandparent, respectively, indicating minimally-degraded RNA. Using RT-PCR SuperScript III Reverse Transcriptase (Thermo-Fisher), a cDNA template was created for the proband, parents, and grandparents. First-strand synthesis was done using a primer near the 3′ end of *MATR3V5* sequence (CCAAATGAAAGTCTGCAAGGCTCA). For PCR, primers were designed to detect *MATR3* variants 1-5. However, no primer pairs produced detectable cDNA.

## Discussion

We present a case of an aggressive form of early-onset FTD. The patient exhibited many of the core features of bvFTD, including behavioral changes, verbal aggression, lack of empathy, stereotyped behaviors, and social withdrawal (15). Brain imaging showed significant atrophy in the frontal and anterior temporal lobes plus heads of the caudate, as well as hypometabolism maximal in bilateral frontal and temporal lobes and basal ganglia, consistent with bvFTD. This case presents a novel genetic mutation that may be linked with early-onset bvFTD. In the patient, one parent, and one grandparent, we discovered an insertion of *MATR3V5* cDNA with flanking 15 bp repeats. However, second- and third-degree relatives were asymptomatic. The clinical symptoms observed in this case do not align with existing mutations in *MATR3* associated with early-onset bvFTD, most of which are point mutations (16). This case is unique because of the patient's atypical clinical presentation as early-onset bvFTD associated with a mechanism of mutation that has not been linked to bvFTD.

The insertion into chromosome 12 of the *MATR3V5* transcript likely occurred through retrotransposition. *MATR3V5* is a rare splice variant of the *MATR3* gene native to chromosome 5 (17). Some point mutations in *MATR3*—S85C, F115C, and T622A—are associated with FTD-ALS spectrum pathology as well as distal myopathy and pharyngeal weakness (17, 18), but retrotransposition of *MATR3V5* has, to the best of our knowledge, never been associated with bvFTD. Since *MATR3* mutations have previously been linked to FTD-ALS spectrum, it is possible that the novel *MATR3* retrotransposition found in this case is causally linked to the observed neurodegeneration.

Often, retrotransposition is benign and associated with evolutionary genetic expansion and gene regulation (19). However, disruptive retrotransposition has been linked to neurological diseases such as Aicardi-Goutières syndrome, multiple sclerosis, ALS, and Alzheimer's disease (20, 21). For example, increased levels of TDP-43, the most common pathological biomarker in FTD-ALS spectrum, leads to loss of post-transcriptional gene silencing responsible for retrotransposable element (RTE) repression (21, 22). Increased activity of RTEs in TDP-43 mediated diseases leads to disruptive retrotransposition, indicated by DNA damage/DNA nicking, and subsequently results in apoptosis (21). Although disruptive retrotransposition has been observed in FTD-ALS, the *MATR3* retrotransposition in the proband's case is unlikely to be a consequence of neurodegeneration since it is present in healthy carriers.

In most cases of FTD-ALS, TDP-43 is the primary component of ubiquitinated-positive cytoplasmic inclusions where the C-terminal fragments of TDP-43 are ubiquitinated and phosphorylated (5, 21, 22). In HEK293-FT cells, endogenous TDP-43 has been shown to co-immunoprecipitate with MATR3 protein (16). Further, the S85C mutant of MATR3 showed increased binding to TDP-43 compared to wild-type MATR3, but this effect was not seen in F115C and T622A mutants (16). Investigation of TDP-43's interaction with MATR3 may shed light on the mechanisms of FTD-ALS pathogenesis, though it remains unclear how retrotransposition of *MATR3V5* into CH12, and potential aberrant expression of *MATR3V5*, impacts cellular function.

In a small number of FTD-ALS cases, FUS, another DNA/RNA binding protein, is the primary pathological protein involved (23). A missense mutation in the *FUS* gene has recently been identified as a cause of familial ALS. A mutation in FUS has also been linked to tau-negative and TDP-43-negative FTLD subtypes (13). In addition, mutations in FUS have been associated with MATR3 and TDP-43 cytoplasmic aggregation (24, 25). Complex interactions amongst proteins implicated in FTD-ALS indicate numerous pathways by which MATR3 dysfunction may cause cognitive deficits. Further study of MATR3's interactome is crucial to understanding its mechanism in FTD.

MATR3 modulation emulates aspects of FTD-ALS pathology *in vitro*. Elevated levels of wild-type MATR3 and MATR3 mutants (S85C, F115C, P154S, and T622A) elicit a dose-dependent toxicity in primary rat mixed cortical cultures. Similarly, siRNA knockdown of endogenous *MATR3* also results in greater cell death compared to non-targeting siRNA. These results illustrate that both increased and decreased neuronal MATR3 leads to increased cell death, exemplifying that the mechanism of MATR3 in FTD-ALS pathology may be a gain or loss of function, or both (11).

It is unclear whether the *MATR3V5* retrotransposition on CH12 encodes an mRNA or produces a protein. *MATR3* expression in the blood is low, and attempts to isolate mRNA or protein from blood and serum samples have been largely unsuccessful. Transcription of *MATR3V5* from CH12 could conceivably increase or decrease MATR3 protein levels by subsequent translation or RNA-silencing, respectively. *MATR3V5* mRNA from CH5 codes for the MATR3 isoform b protein. Isoform a, translated from variants 1-4, is 847 amino acids in length while, isoform b translated from variant 5, is 559 amino acids and shorter on the N terminus. Both isoforms are DNA/RNA binding proteins and carry the same main domains (11, 26). Determination of the functional outcome of CH12-*MATR3V5* transcription requires protein-level analysis.

In future studies, it would be informative to culture forebrain neurons with this mutation using patient derived induced pluripotent stem cells (iPSCs), as this provides a more direct means to study differential localization, altered transcription and translation levels, and mechanisms of MATR3. Notably, *MATR3V5* is located 20 kbp downstream of the *LHX5* gene on CH12. *LHX5* encodes a protein family that function as transcriptional regulators of neuronal differentiation, migration, and development of the forebrain. Because of its chromosomal proximity to a gene active in the cortex and hippocampus, it will be worth studying how the *MATR3V5* insertion may affect—or be impacted by—the *LHX5* locus. In addition, MATR3 cellular localization can be studied using iPSCs. MATR3 localization has been shown to play a role in neurotoxicity. Cytoplasmic MATR3 redistribution from the nucleus extends survival in neurons that overexpress MATR3, suggesting that nuclear MATR3 mediates neurotoxicity (11). Epigenetic differences between the patient and the unaffected carriers could also result in differences in expression of the *MATR3* retrotransposed gene. Collectively, these analyses will allow a thorough investigation of the MATR3 protein and transcript and its role in the pathology of early-onset FTD.

This case study highlights a novel gene retrotransposition of *MATR3V5* associated with a rare case of early-onset bvFTD. The results of this case study illustrate that point mutations may not be the only genetic mechanism by which *MATR3* contributes to FTD. These findings also add to our knowledge of potentially active pseudogenes contributing to dementia pathologies. Future studies will investigate the significance of this gene retrotransposition, potential role of *MATR3V5* in disease, and its association with the early-onset bvFTD pathology presented in this case. Moreover, a thorough understanding of this novel *MATR3* mutation may help elucidate mechanisms of matrin-3 dysregulation in other neurodegenerative contexts.

## Data Availability Statement

The raw data supporting the conclusions of this article will be made available by the authors, without undue reservation.

## Ethics Statement

This study was approved by the University of Minnesota's institutional review board. All procedures performed during this study involving human participants were in accordance with the ethical standards of the institutional review board at the University of Minnesota and with the 1964 Helsinki Declaration and its later amendments or comparable ethical standards. Surrogate consent was received for the participant. Informed consent was obtained from all other individuals able to independently consent. Written informed consent was obtained from the participant's authorized representative for the publication of any potentially identifiable images or data included in this article.

## Author Contributions

MC and KV obtained IRB approval. MC, NV, and KV drafted the manuscript. KV and BB examined the patient. DD, MB, and MK completed the genetic analysis. NV completed the PCR assays. SP provided guidance and technical support for PCR assays. All authors reviewed and revised the final manuscript.

## Conflict of Interest

The authors declare that the research was conducted in the absence of any commercial or financial relationships that could be construed as a potential conflict of interest.
